# Study on HPLC fingerprint of *Saposhnikovia divaricata* lipophilic components and anti-aging effects

**DOI:** 10.1038/s41598-024-75824-0

**Published:** 2024-10-28

**Authors:** Xianwen Yue, Yang Xu, Bin Sun, Yali Shang, Zhuo Yu, Yingming Hu, Huiwei Bao, Hao Yue

**Affiliations:** 1 Jilin Ginseng Academy, Changchun University of Chinese Medicine, Changchun, 130117 China; 2 School of Pharmacy, Changchun University of Chinese Medicine, Changchun, 130117 China; 3 School of Pharmacy, Baicheng Medical College, Baicheng, 137000 China

**Keywords:** *Saposhnikovia divaricata* lipophilic components, HPLC fingerprint, Chemical pattern recognition, Content determination, Anti-aging effects, Pharmacodynamics, Analytical chemistry

## Abstract

*Saposhnikovia divaricata* is commonly used in clinical practice as a heat-clearing and detoxifying traditional Chinese medicine. Current research on *Saposhnikovia divaricata* has mostly focused on chromones, while studies on its lipophilic parts remain scarce. In this study, samples of the lipophilic components of *Saposhnikovia divaricata* (SDL) from different regions in China were collected to establish an HPLC fingerprint. The quality of SDL was evaluated through chemical pattern recognition and multi-component content determination. Furthermore, using the Caenorhabditis elegans (C. *elegans*) model, the anti-aging ability of SDL was preliminarily studied. In the HPLC fingerprints of 11 batches of SDL, 19 common peaks were identified, and 4 components were characterized as follows: peak 14, linolenic acid (ALA); peak 15, docosahexaenoic acid (DHA); peak 16, linoleic acid (LA); peak 17, oleic acid (OA). The similarity of the fingerprint ranged from 0.704 to 0.929. The results indicate that the content of ALA, DHA, LA, and OA are 0.040–0.440 mg/g, 0.003–0.116 mg/g, 1.047–6.460 mg/g, and 0.040–0.294 mg/g, respectively. HCA analysis divided 11 batches of SDL into two categories, and PCA analysis extracted 6 principal components with a cumulative contribution rate of 85.91%. The anti-aging ability of the SDL was proved by measuring the lifespan, reproductive capacity, body length, mobility, lipofuscin content, and heat stress resistance of C. *elegans*. The combination of high-performance liquid chromatography fingerprint and chemical pattern recognition for multi-component content determination is a reliable, comprehensive, and promising evaluation of the quality of SDL. The confirmation of SDL’s anti-aging ability provides the possibility for the research of *Saposhnikovia divaricata* in the field of healthy food and cosmetics.

## Introduction

*Saposhnikovia divaricata*, with a history of over 2000 years in traditional Chinese medicine clinical practice, is a medicinal herb widely produced in various regions of China such as Heilongjiang, Jilin, Liaoning, Hebei, Shandong, and Inner Mongolia^1^. *Saposhnikovia divaricata* has a wide range of effects and is recorded as a superior herb in the ancient book “Shennong Bencao Jing“^2^. Its traditional functions include dispelling wind, relieving surface symptoms, eliminating dampness, alleviating pain, and relieving spasms^3^. Studies have discovered that *Saposhnikovia divaricata* contains chemical components such as chromones, polysaccharides, volatile oils, and coumarins^4,5^. These components exhibit pharmacological activities such as antipyretic, anti-inflammatory, analgesic, and anti-tumor effects^6–9^.

In recent years, there has been abundant research on *Saposhnikovia divaricata*. Zhang et al.^10^ conducted a study on the quality of standard decoctions of *Saposhnikovia divaricata*. They quantitatively analyzed four components, namely ligustilide glycoside, ligustilide, 5-O-methylvisamminol glycoside, and ferulic acid glycoside, in 15 batches of standard decoctions of *Saposhnikovia divaricata* slices. They found that the content of these four components can be affected by the origin of the herb. Qiu et al.^11^ used a multi-component quantitative analysis method to compare the content of the above four components in wild and cultivated *Saposhnikovia divaricata*. They discovered that the content of ligustilide, 5-O-methylvisamminol glycoside, and ferulic acid glycoside in wild *Saposhnikovia divaricata* was significantly higher than that in cultivated *Saposhnikovia divaricata*, while there was no significant difference in the content of ligustilide glycoside between the two. Most of these quality standard studies mainly focused on chromone components, with some involvement of volatile oil components^12–14^ and polysaccharide components^15^. However, as a medicinal herb from plants, the lipophilic components commonly present in *Saposhnikovia divaricata* cannot be ignored. Fatty acids not only provide energy for metabolic processes but also possess a wide range of pharmacological activities, including anti-inflammatory, anti-tumor effects, effects on the central nervous system, regulation of blood lipids, antioxidation, and scavenging of free radicals^16–19^.

Caenorhabditis elegans (C. *elegans*), commonly known as the elegant or nematode worm, has been widely used in aging and neurodegenerative disease research due to its advantages such as a fully known genome, clear genetic background, low cost, short lifespan, rapid reproduction with high offspring numbers, rich genetic resources, stable behavioral response patterns, and sensitive and reliable results^20–23^. The signs of aging in worms are evident, including shortened body length, intestinal atrophy, gonad degeneration, decreased motility (pharyngeal pumping rate, pharyngeal contraction), and reduced reproductive capacity, providing abundant observation indicators for experiments. Therefore, C. *elegans* serves as a classic aging model for anti-aging studies of traditional Chinese medicine^24^. The main manifestations include the gradual shrinking of individual worms, overall wrinkling, and eventually a decrease in motility. The quantity and quality of worm eggs decline as well. The endogenous autofluorescent substance lipofuscin mainly accumulates in aging cells such as neurons, myocardium, and liver tissues, and is therefore referred to as “age pigment”^25^. These indicators can be used to evaluate the efficacy of drugs in delaying the aging process of C. *elegans*.

**Fig. 1:**
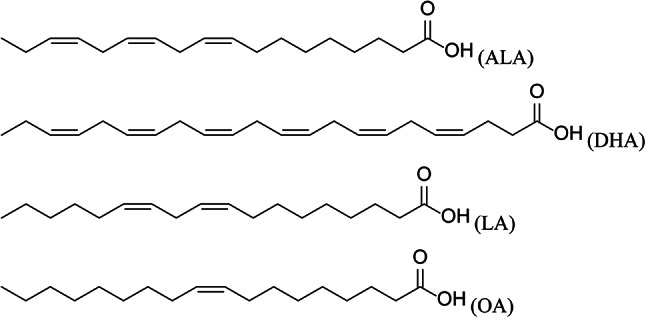
Four chemical structural formulas of natural fatty acids.

Synthesis of the above content, this study focuses on the lipophilic components of *Saposhnikovia divaricata* (SDL) to investigate its quality and determine the content of four natural fatty acids, linolenic acid (ALA), docosahexaenoic acid (DHA), linoleic acid (LA) and oleic acid (OA) (see structural formula in Fig. 1), thereby improving the quality standards of *Saposhnikovia divaricata*. Additionally, through the C. *elegans* aging model, the anti-aging ability of SDL is preliminarily studied, laying a foundation for research on *Saposhnikovia divaricata* in the field of anti-aging.

## Materials and methods

### Materials and reagents

The dried roots of *Saposhnikovia divaricata* (Turcz.) Schischk. from the Umbelliferae family, met the quality requirements outlined in the “Pharmacopoeia of the People’s Republic of China” 2020 edition. The fresh samples were collected from 8 regions in China, including Inner Mongolia, Hebei, Shanxi, Jilin, Sichuan, Anhui, Gansu, and Heilongjiang. The specific place of origin is shown in Table 5. The plants were identified by Associate Professor Jiaming Xu, at the College of pharmacy, Changchun University of Chinese Medicine, China. All samples are preserved in the Herbarium of the School of Pharmacy, Changchun University of Chinese Medicine, with voucher numbers B2022120201-B022120218. The Saposhni-kovia divaricata samples collected in this study are non wild plants and artificially planted plants. We comply with the IUCN Policy Statement on Research Involving Species at Risk of Extinction and the Convention on the Trade in Endangered Species of Wild Fauna and Flora.

The control substance information in this paper is as follows: linolenic acid (9,12,15-Octadecatrienoic Acid, Lot No. 21090323, Purity 99.0%), docosahexaenoic acid (cis-4,7,10,13,16,19-Docosahexaenoic Acid, Lot No. 21081279, Purity 99.1%), linoleic acid (cis-9,12-Octadecenoic Acid, Lot No. 21090749, Purity 98.9%), oleic acid (cis-9-Octadecenoic Acid, Lot No. 21081197, Purity 99.6%), acquired from Tan-Mo Technology Co., Ltd. Acetonitrile (Chromatographically Pure, Thermo Fisher Scientific (China) Co., Ltd.); Tetrahydrofuran (Analytically Pure, Fu Chen (Tianjin) Chemical Reagent Co., Ltd.); Distilled water (Hangzhou Wahaha Group Co., Ltd.); other reagents were all of analytical grade.

Wild-type C. *elegans* (N2 strain), Escherichia coli, were donated by the Institute of Translational Medicine, Qingdao University; potassium dihydrogen phosphate, dipotassium hydrogen phosphate, magnesium sulfate, sodium hydroxide, purchased from Guangdong Guanghua Technology Co., Ltd.; calcium chloride, purchased from Xilong Science Co., Ltd.; cholesterol, purchased from Shanghai Maclin Biochemical Technology Co., Ltd.; streptomycin, purchased from Shandong Lukang Pharmaceutical Co., Ltd.; agar powder, purchased from Tianjin Guangfu Technology Development Co., Ltd.; peptone, purchased from Beijing Aobostar Biotechnology Co., Ltd.; sodium hypochlorite, purchased from Chengdu Kelong Chemical Co., Ltd.; M9 buffer, NGM medium, LB medium, and worm lysate were prepared according to conventional methods^26^.

### Preparation of reference solution

During the experiment, an AB135-S electronic analytical balance (Mettler-Toledo International Inc.) was used to weigh each standard. An appropriate amount of docosahexaenoic acid reference substance was taken, accurately weighed, dissolved in tetrahydrofuran, and diluted to 5 mL, resulting in a concentrated solution of docosahexaenoic acid reference substance with a concentration of 2.85 mg/mL. Then, an appropriate amount of linoleic acid, and oleic acid reference substances were taken, accurately weighed, and precisely transferred 0.5 mL of the aforementioned docosahexaenoic acid reference solution into a 5 mL volumetric flask. Tetrahydrofuran was added to dissolve the mixture, and the solution was diluted to the mark, resulting in a mixed reference solution (a) containing 2.914 mg of linoleic acid, 0.285 mg of docosahexaenoic acid, and 2.012 mg of oleic acid per 1 mL. An appropriate amount of linolenic acid was taken, accurately weighed, and transferred into a 10 mL volumetric flask. Then, 1 mL of the mixed reference solution (a) was precisely transferred, tetrahydrofuran was added to dissolve the mixture, and the solution was diluted to the mark, resulting in a mixed reference solution containing 0.2914 mg of linoleic acid, 0.0285 mg of docosahexaenoic acid, 2.138 mg of linolenic acid, and 0.2012 mg of oleic acid per 1 mL.

### Preparation of test solution

An appropriate amount of *Saposhnikovia divaricata* sample was taken, pulverized, and approximately 10 g of powdered sample was weighed using a DT series electronic balance (Yiou Instruments Co., Ltd., Changshu, Jiangsu Province). The sample powder was placed in a stoppered conical bottle, and 50 mL of petroleum ether (60–90 °C) was added. The bottle was sealed, shaken, and weighed. Ultrasonication (power: 250 W, frequency: 40 kHz, provided by Kunshan Ultrasonic Instruments Co., Ltd.) was performed for 30 min, followed by standing at room temperature. The weight was measured, and the weight was adjusted. The mixture was shaken, filtered, and the extraction process was repeated twice. The filtrates were combined, and the solvent was recovered using an R series rotary evaporator (Shanghai Sensheng Technology Co., Ltd.). Then, an appropriate amount of tetrahydrofuran was added to transfer the mixture and diluted to a 10 mL volumetric flask. The solution was shaken, filtered through a 0.22 μm microporous membrane, and the test solution was obtained.

### Chromatographic conditions

The *Saposhnikovia divaricata* sample was analyzed using a 1260 high-performance liquid chromatography system (Agilent Technologies, USA). The chromatographic column used was a Diamonsil™ C_18_ column (250 mm × 4.6 mm, 5 μm). The mobile phase consisted of acetonitrile (A) and 0.2% phosphoric acid aqueous solution (B), using a gradient elution method (0–15 min, 40% A; 15–20 min, 40%→50% A; 20–35 min, 50%→53% A; 35–40 min, 53%→60% A; 40–60 min, 60%→80% A; 60–85 min, 80%→90% A; 85–95 min, 90%→100% A). The detection wavelength was set at 205 nm. The flow rate was 1.0 mL/min. The column temperature was maintained at 30 °C. The injection volume was 5 µL.

### Fingerprint study

#### Methodological investigation of fingerprint analysis

##### Precision test

Four microliters of the same reference solution were accurately aspirated and injected six times under the aforementioned chromatographic conditions. Using linolenic acid peak 16 as the reference peak, the relative retention time and relative peak area of each common chromatographic peak were calculated for RSD.

##### Stability test

Four microliters of the same test solution were accurately aspirated and analyzed at 0, 2, 4, 6, 8, and 12 h under the aforementioned chromatographic conditions. Using linolenic acid peak 16 as the reference peak, the relative retention time and relative peak area of each common chromatographic peak were calculated for RSD.

##### Repeatability test

Six samples of *Saposhnikovia divaricata* (Sample S1) were prepared according to the method described in “Content of ALA, DHA, LA, and OA in SDL” and analyzed under the chromatographic conditions described in “ Study on the anti-aging effect of SDL on C. *Elegans*”. Using linolenic acid peak 16 as the reference peak, the relative retention time and relative peak area of each common chromatographic peak were calculated.

#### Establishment of fingerprint and similarity analysis

Analyze 11 batches of SDL solutions according to the method described in Section “Chromatographic conditions” to obtain HPLC spectra. Then, import the data into the “Chinese Medicine Chromatographic Fingerprint Similarity Evaluation System (Chinese Pharmacopoeia Committee, 2012 Edition)” to establish HPLC fingerprint spectra and conduct similarity analysis.

### Chemical pattern recognition

The software SPSS (SPSS USA, version 20.0) and Origin Pro (2019b) were used for the principal component analysis (PCA)and hierarchical cluster analysis (HCA) to evaluate the quality of SDL.

### Content determination

#### System adaptability and methodological investigation

##### Linearity study

Precision-weighed reference standards including 7.42 mg of linoleic acid, 5.88 mg of docosahexaenoic acid, 34.40 mg of oleic acid, and 12.53 mg of linolenic acid were placed in a 5 mL volumetric flask, dissolved in tetrahydrofuran and made up to volume, shaken well to obtain the mixed standard solution A. Portions of mixed standard solution A were precisely pipetted at volumes of 0.01 mL, 0.1 mL, 0.5 mL, 1 mL, and 2 mL, diluted with tetrahydrofuran to 5 mL to obtain mixed standard solutions B-F. The mixed standard solutions A-F were analyzed according to the chromatographic conditions in Section “Chromatographic conditions”, and the peak areas were recorded.

##### Precision test

A sample solution from sample S1 was consecutively injected six times under the chromatographic conditions described in this paper, and the peak areas were recorded.

##### Stability test

A sample solution from sample S1 was injected at 0, 2, 6, 12, 24, and 48 h after preparation under the chromatographic conditions described in this paper, and the peak areas were recorded.

##### Repeatability test

Approximately 10 g of sample S1 powder was weighed and divided into 6 portions. Following the methods in Section “Preparation of test solution”, 5 µL of the sample solution was injected for analysis under the chromatographic conditions described in this paper, and the peak areas were recorded to calculate the content.

##### Recovery test

Six portions of sample S1 powder with known contents, each weighing about 5 g, were added with 0.1 ml of linoleic acid reference solution (5.69 mg/mL), 0.05 ml of docosahexaenoic acid reference solution (0.285 mg/mL), 1 ml of oleic acid reference solution (8.792 mg/mL), and 0.1mL of linolenic acid reference solution (3.094 mg/mL). These were then processed according to the method in Section “Preparation of test solution” and analyzed under the chromatographic conditions in Section “Chromatographic conditions”, and the recovery rates were calculated.

#### Sample content determination

Eleven batches of *Saposhnikovia divaricata* samples were collected. Sample solutions were prepared according to the method in Section “Preparation of test solution”, and then determined the content of linoleic acid, docosahexaenoic acid, oleic acid, and linolenic acid under the chromatographic conditions in Section “Chromatographic conditions”.

### Study on the delayed aging effect of SDL on C. *Elegans*

#### Cultivation and synchronization of C. *Elegans*, preparation of medicinal solution

C. *elegans* were rinsed with M9 solution into a 1.5 mL centrifuge tube, centrifuged at 3000 rpm for 3 min at 4 °C, and the supernatant was removed. This process was repeated twice. The worms were then synchronized using the hypochlorite method. Sequentially, 200 µL of sodium hypochlorite and 100 µL of sodium hydroxide were added in the dark, quickly covered, shaken for 90 s, and then made up to 1.5 mL with M9 at 4 °C and centrifuged at 5000 rpm for 1 min to remove the supernatant. This was followed by adding 1.5 ml of M9, centrifuging at 3000 rpm for 3 min at 4 °C, and repeating this washing step twice to remove the lysate. The worms at the bottom of the centrifuge tube were aspirated using a pipette and placed on NGM agar plates, and then placed in a constant temperature incubator at 20 °C for about 48 h to allow the worms to grow to the L4 stage for subsequent drug treatment.

Prepare the SDL (S1, Inner Mongolia 202210) according to the method described in Section “Preparation of test solution”. After recovering the solvent, add an appropriate amount of dimethyl sulfoxide (DMSO) to dissolve and obtain SDL mother liquor. Dilute the SDL mother liquor with sterile water and mix it evenly with E.coli OP50 bacterial solution in a 1:1 ratio to obtain SDL solutions with concentrations of 50, 100, and 200 µg/ml, respectively, as LSDL, MSDL, and HSDL groups. The final content of DMSO in each dose is 0.1%. Use a 1:1 mixture of sterile water and E.coli OP50 bacterial solution as the blank group administration solution.

Drop 50 µl of SDL solutions of different concentrations prepared above into fresh NGM culture plates, dry them, and use them for nematode experiments.

#### Lifespan determination of nematodes

The experiment included a blank group and experimental groups LSDL, MSDL, and HSDL. The blank group consisted of nematodes cultured on NGM medium without any extract, while the experimental groups were cultured on NGM medium containing different concentrations of SDL (50 µg/mL, 100 µg/mL, and 200 µg/mL). Synchronized hermaphrodite and male nematodes at the L4 stage were transferred to the corresponding NGM medium. The day of transfer was marked as 0d, and every 24 h, the nematodes were transferred to new culture dishes of the respective experimental groups, each board should have no less than 10 worms, with 3 boards per group. The number of deaths and survivors were recorded daily until all the nematodes died.

#### Reproductive capacity determination

L4 stage nematodes were placed on NGM plates of the blank group and NGM plates containing different concentrations of SDL (50 µg/mL, 100 µg/mL, and 200 µg/mL) (1 worm per plate, with 6 replicates). The day the nematodes started laying eggs was marked as day 1, and egg counting was performed every 24 h. The nematodes were transferred to new culture dishes. The experiment lasted for 5 days, and the total number of eggs laid each day was recorded.

#### Nematode body length measurement

Synchronized L4 stage nematodes were transferred to the blank group and NGM medium containing different concentrations of SDL (50 µg/mL, 100 µg/mL, and 200 µg/mL). After growing in the incubator for 5 days, the nematodes were washed and transferred to microscope slides using M9 buffer solution. The nematodes’ body lengths were measured under a microscope. Each group had 10 nematodes with 3 replicates.

#### Locomotion ability determination

Synchronized L4 stage nematodes were collected and treated with SDL at concentrations of 50 µg/mL, 100 µg/mL, and 200 µg/mL. Six nematodes were placed in each culture dish, with 3 replicates per group. The locomotion ability of the nematodes on the 5th and 10th day of the culture period was observed and recorded. (One complete forward and backward movement of the nematode’s head and tail was counted as 1 body swing. Each nematode’s movements were recorded for 30 s).

#### Lipofuscin content determination

As nematodes age, their intestines produce a self-fluorescent pigment called lipofuscin. Adult nematodes treated with SDL at concentrations of 50 µg/mL, 100 µg/mL, and 200 µg/mL for 8 days or left untreated were anesthetized with levamisole (Beijing Chemical Factory, China), fixed on a 2% agarose pad, and their lipofuscin levels were measured. The fluorescence intensity was observed using a BX63 fluorescence microscope (Olympus, Japan), and fluorescent images of different groups were captured. Each culture dish had no fewer than 15 worms, with 3 replicates per group.

#### Heat stress assay

Nematodes at the L4 stage, after synchronization, were subjected to different drug interventions for 5 days and then transferred to a blank NGM plate and placed in a 35 °C incubator. The survival of the nematodes was observed and recorded every hour until all nematodes died. Each board should have no less than 10 worms, with 3 boards per group.

### Data analysis

The results were analyzed using Microsoft Excel software, SPSS 26.0 software, and one-way analysis of variance (ANOVA), with a significance level set at *P* < 0.05. The reliability of survival curves was analyzed using GraphPad Prism 8.02 software.

## Results and discussion

### Fingerprinting study

#### Methodological Investigation

The analytical method for determining SDL content was validated according to the “Guidelines for Validation of Analytical Methods” in the Pharmacopoeia of the People’s Republic of China. Using linoleic acid (LA) as the reference peak (Peak 16), in the precision test, the relative standard deviation (RSD) of the retention time for each common chromatographic peak was less than 1.76%, and the RSD of the peak area was less than 2.17%, indicating good precision of the instrument. In the stability test, the RSD of the retention time for each common chromatographic peak was less than 1.93%, and the RSD of the peak area was less than 2.59% within 12 h, indicating good stability of the test solution. In the repeatability test, the RSD of the retention time for each common chromatographic peak was less than 2.04%, and the RSD of the peak area was less than 2.83%, indicating good repeatability of the method.

#### Establishment of fingerprint and similarity evaluation

**Fig. 2:**
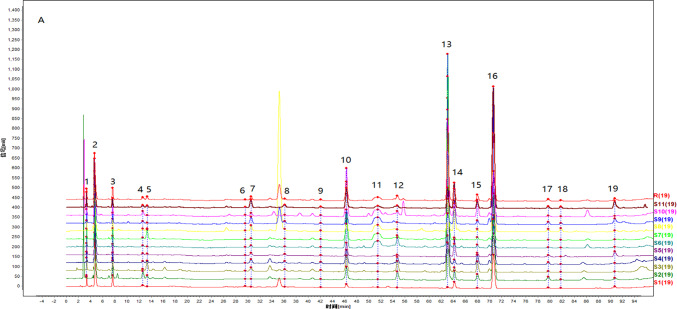
HPLC fingerprint of SDL samples from different regions. By comparing with the reference solution, peaks 14–17 were identified as ALA, DHA, LA, and OA, respectively.

Eleven batches of SDL solutions were taken for analysis. Using the chromatogram of sample S1 as the reference fingerprint, multi-point calibration was performed to generate overlay and control fingerprints (see Fig. 2). A total of 19 common peaks were identified, and through comparison with reference standards, four components were identified: Peak 14 as linolenic acid (ALA), Peak 15 as docosahexaenoic acid (DHA), Peak 16 as linoleic acid (LA), and Peak 17 as oleic acid (OA). The retention time of each shared peak is shown in Table 1. Using the reference peak 16 (LA), the relative retention time RSD values of the common peaks ranged from 0.05 to 1.27%, and the relative peak area RSD values ranged from 36.55 to 195.02%, indicating that different batches of samples had similar chemical composition but significant differences in content. The similarity evaluation results can be found in Table 2, where the similarity values of samples from different regions ranged from 0.704 to 0.929. This indicates that the quality of SDL samples from different regions is relatively stable, with similar chemical composition but varying content. The similarity values of samples from Inner Mongolia (S1) and Gansu (S9) were relatively low, while the similarity values of samples from other regions were relatively high, all being > 0.805.

**Table 1:** Retention time of common peaks and corresponding known compounds.

No.	Retention time	Compound no.	Retention time	Compound
1	3.361	11	51.516	
2	4.628	12	54.740	
3	7.639	13	63.062	
4	12.641	14	64.165	ALA
5	13.335	15	67.974	DHA
6	29.548	16	70.632	LA
7	30.535	17	79.706	OA
8	36.087	18	81.772	
9	42.058	19	90.700	
10	46.321	-	-	-

**Table 2:** Similarity evaluation results of SDL fingerprint spectra from different regions.

No.	Similarity	No.	Similarity
S1	0.704	S7	0.815
S2	0.924	S8	0.840
S3	0.892	S9	0.772
S4	0.892	S10	0.929
S5	0.808	S11	0.810
S6	0.805	-	-

### Chemical pattern recognition

#### Hierarchical cluster analysis (HCA)

**Fig. 3:**
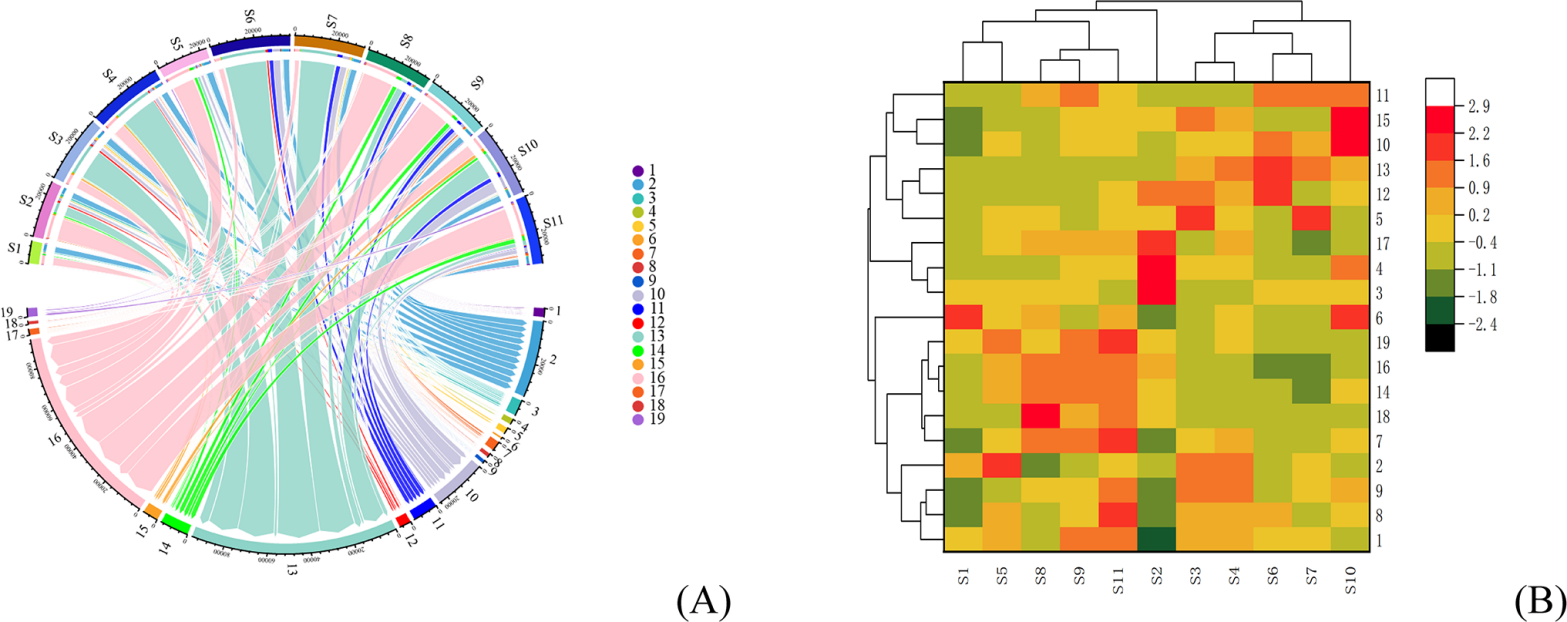
Hierarchical cluster analysis (**A**) Chord plot of common peak area and batch relationship (**B**) Cluster analysis heat map of SDL samples.

The 19 common peaks of 11 batches of SDL were imported into Circlize package and Openxlesx package to generate Fig. 2A. The results showed that the peak areas of each component in origin S1, S5, and S2 were relatively small, and the total active ingredient content was relatively low. Among them, peaks 16 and 13 have the highest proportion of peak area in samples from various production areas, and the two chromatographic peaks are of great significance (see Fig. 3A). Import the common peaks of 11 batches of windproof samples into the Pheatmap package for analysis and plotting, and obtain an 11 × 19 order matrix. Use squared Euclidean distance as the measurement method. The results showed that 11 batches of samples were divided into 2 clusters. Cluster I includes S10, S3, S4, S6, S7; Cluster II includes S1, S8, S9, S11, S2, S5. The heatmap of clustering is shown in Fig. 3B.

#### Principal component analysis(PCA)

**Fig. 4:**
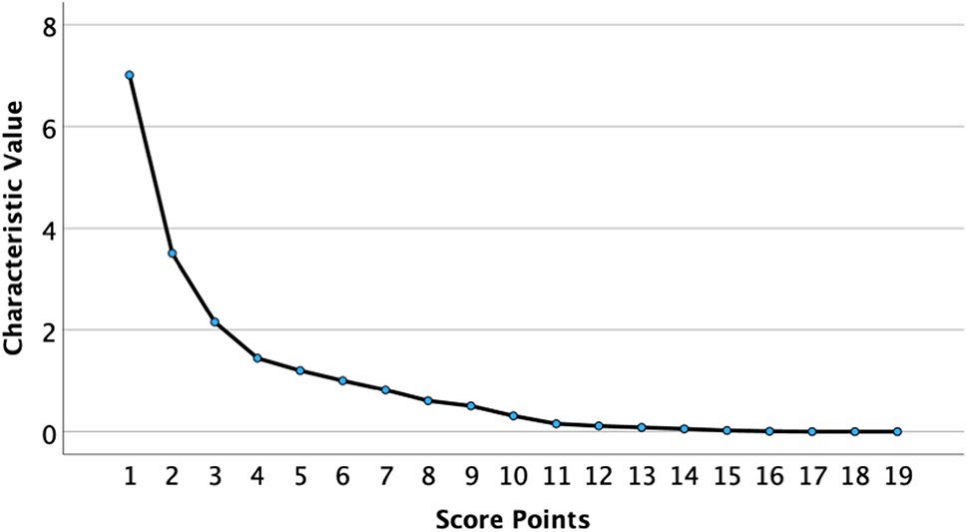
Scree plot.

**Fig. 5:**
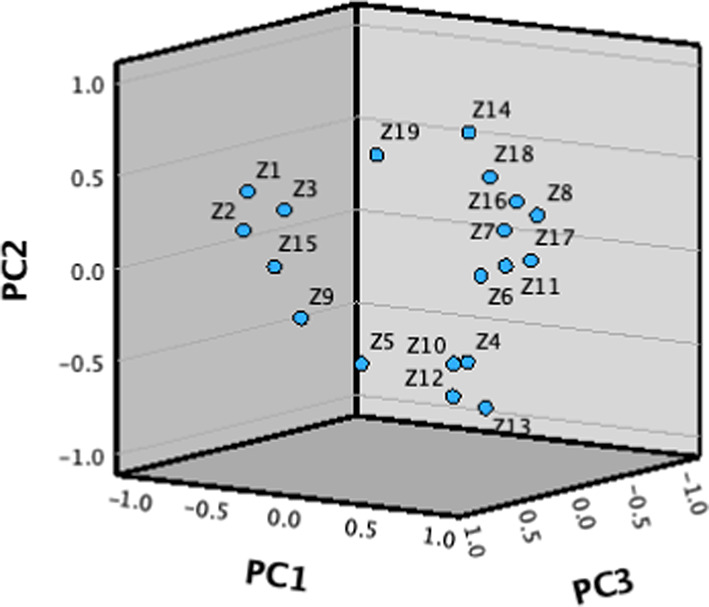
PCA gravel map.

Analyze the differences in SDL chemical composition in different regions through PCA model. In order to ensure the accuracy of sample analysis in different regions, we supplemented 7 batches of *Saposhnikovia divaricata* samples, namely Hebei (Lot No.: 202210), Shanxi (Lot No.: 202210), Jilin (Lot No.: 202210), Sichuan (Lot No.: 202210), Anhui (Lot No.: 202210), Shaanxi (Lot No.: 202211), and Heilongjiang (Lot No.: 202211). Perform factor analysis using the peak areas of 19 common peaks in 18 batches of SDL fingerprint spectra as variables. According to Table 3, based on eigenvalues > 1, six principal components(PC) were obtained with contribution rates of 36.92%, 18.45%, 11.33%, 7.60%, 6.32%, and 5.27%, respectively. The cumulative contribution rate reached 85.91%, indicating that the model has good fitting ability. The first 6 principal components can be used to represent most of the information of the 19 common peaks. The scree plot is shown in Fig. 4, and the principal component analysis is shown in Fig. 5. From the factor loading matrix (Table 4), it can be seen that the information of principal component 1 mainly comes from peaks 1–4, 7, 10–11, 13–18; The information of principal component 2 mainly comes from peaks 5, 12–14, 18, and 19; The information of principal component 3 mainly comes from peaks 8, 9, and 19; The information of principal component 4 mainly comes from peak 11; The information of principal component 5 mainly comes from peaks 5 and 6; The information of principal component 6 mainly comes from peak 8.

**Table 3:** Principal component factor eigenvalues and variance contribution rate.

Initial eigenvalue		
Total	Variance contribution rate %	Cumulative contribution rate %
7.01	36.92	36.92
3.50	18.45	55.37
2.15	11.33	66.70
1.44	7.60	74.30
1.20 1.00	6.32 5.27	80.64 85.91

**Table 4:** Principal component factor load matrix of SDL.

Common peak	PC1	PC2	PC3	PC4	PC5	PC6
1	-0.771	0.348	0.383	-0.035	-0.066	0.137
2	-0.910	0.100	0.219	-0.001	0.075	0.045
3	-0.850	0.175	-0.072	0.056	0.119	-0.223
4	0.525	-0.468	0.199	0.446	-0.230	-0.309
5	-0.148	-0.543	0.217	0.150	0.525	0.209
6	0.386	-0.065	-0.122	0.462	-0.696	0.146
7	0.822	0.284	0.284	0.026	-0.001	0.270
8	0.469	0.213	-0.529	-0.048	0.082	0.585
9	-0.048	-0.184	0.921	0.041	-0.039	0.267
10	0.524	-0.458	0.327	-0.461	-0.279	-0.160
11	0.648	0.037	0.020	-0.695	-0.012	-0.055
12	0.406	-0.671	0.164	0.226	0.296	0.019
13	0.592	-0.718	0.125	-0.263	0.038	0.031
14	0.532	0.771	0.198	-0.065	-0.007	-0.001
15	-0.597	-0.042	0.379	-0.041	-0.260	0.393
16	0.819	0.424	0.164	0.111	0.196	-0.127
17	0.764	0.068	-0.047	0.385	0.262	0.073
18	0.629	0.528	0.136	-0.013	0.044	0.050
19	0.157	0.657	0.512	0.140	0.110	-0.279

### Content of ALA, DHA, LA, and OA in SDL

#### System adaptability test

**Fig. 6:**
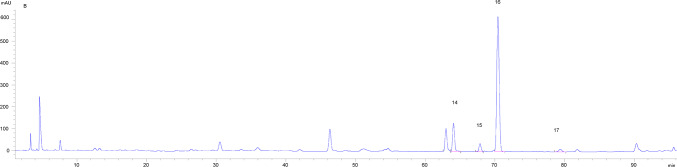
HPLC chromatogram of SDL test solution at 205 nm (14. ALA; 15. DHA; 16. LA; 17. OA ).

Through HPLC analysis, the HPLC chromatogram of SDL was obtained. As shown in Fig. 6, the chromatographic peaks of the components to be tested were completely separated from adjacent peaks, and the peak shapes were symmetrical, with theoretical plate numbers all greater than 3000.

#### Validation of analytical methodology

Using the concentrations of ALA, DHA, LA, and OA as the abscissa (x) and the peak areas as the ordinate (y), linear regression was performed, yielding the following regression equations: ALA y = 5.4183x-47.702, *r* = 0.9996; DHA y = 19.875x-137.26, *r* = 0.9998; LA y = 3.4601x + 197.5, *r* = 0.9996; OA y = 1.8665x + 0.0544, *r* = 0.9997. The results indicate that there is a good linear relationship between the concentrations of ALA (2.968~1484 µg/mL), DHA (2.352~1176 µg/mL), LA (13.76~6880 µg/mL), OA (5.012~2506 µg/mL), and the peak areas.

In the precision test, the RSD values of the peak areas for ALA, DHA, LA, and OA were 1.02%, 0.63%, 1.01% and 0.89% respectively, indicating good instrument precision. In the stability test, the RSD values of the peak areas for ALA, DHA, LA, and OA were 1.32%, 1.07%, 1.16% and 1.64% respectively, within 48 h, indicating good stability of the processed samples. In the repeatability test, the average contents of ALA, DHA, LA, and OA in the samples were 0.130, 0.004, 1.804 and 0.068 mg/g, with RSD values of 1.72%, 1.49%, 1.32% and 1.39%, respectively, indicating good repeatability of the sample processing method. In the recovery test, the average recovery rates of ALA, DHA, LA, and OA were 98.03%, 98.83%, 99.02%, and 99.10%, with RSD values of 1.33%, 1.93%, 1.56%, and 1.90%, respectively.

#### Determination of content

The 11 batches of *Saposhnikovia divaricata* samples collected were processed and analyzed according to the content determination experimental method in this study to calculate the content of ALA, DHA, LA, and OA in the samples, as shown in Table 5. The results indicate that the content of ALA, DHA, LA, and OA are 0.040–0.440 mg/g, 0.003–0.116 mg/g, 1.047–6.460 mg/g, and 0.040–0.294 mg/g, respectively.

**Table 5:** Multi components content in 11 batches of SDL (mg/g).

No.	Origin	Lot no.	ALA	DHA	LA	OA
S1	Inner Mongolia	202,210	0.116	0.003	1.761	0.064
S2	Inner Mongolia	202,211	0.166	0.046	4.601	0.294
S3	Hebei	202,211	0.080	0.075	1.950	0.096
S4	Shanxi	202,210	0.086	0.067	2.479	0.196
S5	Jilin	202,211	0.314	0.026	4.639	0.141
S6	Sichuan	202,210	0.061	0.020	1.159	0.062
S7	Anhui	202,211	0.040	0.018	1.047	0.040
S8	Gansu	202,211	0.413	0.018	6.460	0.203
S9	Gansu	202,210	0.430	0.027	6.316	0.199
S10	Shanxi	202,211	0.251	0.116	2.336	0.082
S11	Heilongjiang	202,211	0.440	0.047	6.173	0.195

###  Study on the anti-aging effect of SDL on C. *Elegans*

#### Lifespan determination of C. *Elegans*

**Fig. 7:**
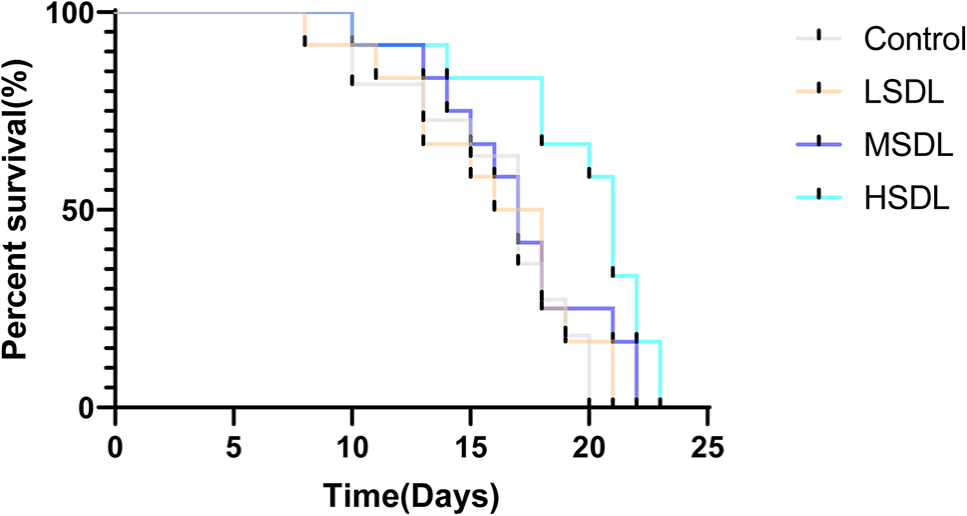
Effects of SDL on the lifespan of *C. elegans*.

The lifespan of C. *elegans* is a crucial indicator of aging^27^. As per the results in Table 6; Fig. 7, the groups treated with 50 µg/mL, 100 µg/mL, and 200 µg/mL of SDL all showed an extension of lifespan in C. *elegans*, with lifespan curves shifting to the right. The longevity extension effect of 200 µg/mL of SDL was the most significant (*P* < 0.05), with an average lifespan increase of 19.44% and the longest lifespan reaching 22.33 ± 1.15 days. It can be inferred that SDL have the effect of extending the lifespan of C. *elegans*.

**Table 6:** Statistical analysis of *C. Elegans* survival time.

Group	Dose (µg/mL)	The average lifespan /d	Increase percentage /%	The longest lifespan /d
Control	-	14.86 ± 0.40	-	19.67 ± 0.58
LSDL	50	15.44 ± 0.45	3.93	21.00 ± 1.00
MSDL	100	16.22 ± 0.52	9.16	21.33 ± 0.58
HSDL	200	17.75 ± 1.23*	19.44	22.33 ± 1.15

*P < 0.05, ** *P* < 0.01 and *** *P* < 0.001 compared with Control group.

#### Reproductive capacity

**Fig. 8:**
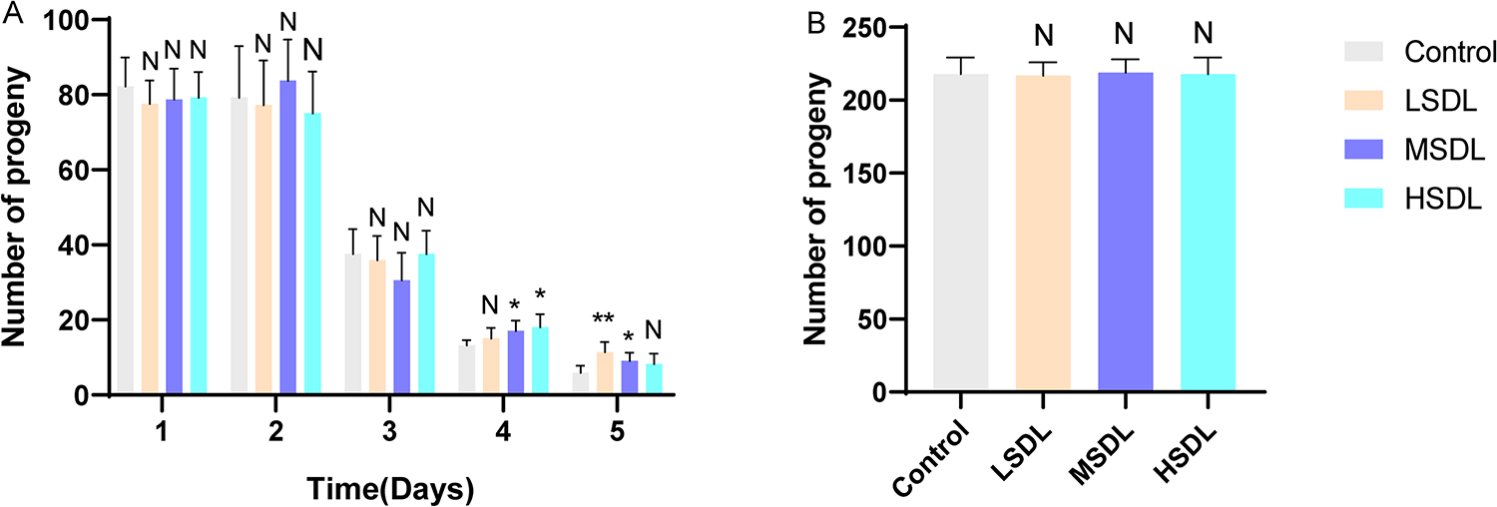
The reproductive effects of SDL on *C. elegans* (**A**) the daily egg production within 5 days (**B**) the total egg production within 5 days. ^N^*P*>0.05, *P < 0.05 and ** *P* < 0.01 compared with Control group.

Starting from the L4 stage, the impact of SDL on the lifespan extension of C. *elegans* on offspring reproduction was investigated. Because reducing the egg-laying of C. *elegans* can extend lifespan. C. *elegans* starts laying eggs from the L4 stage, with higher egg production in the first 3 days. As shown in Fig. 8, the daily egg production within 5 days varied in the groups treated with SDL, but when considering the total egg production, there was no significant difference among the groups. Therefore, these data indicate that the lifespan extension effect of SDL on C. *elegans* is a direct impact and not achieved by inhibiting the reproduction of C. *elegans*.

#### Body length measurement

**Fig. 9:**
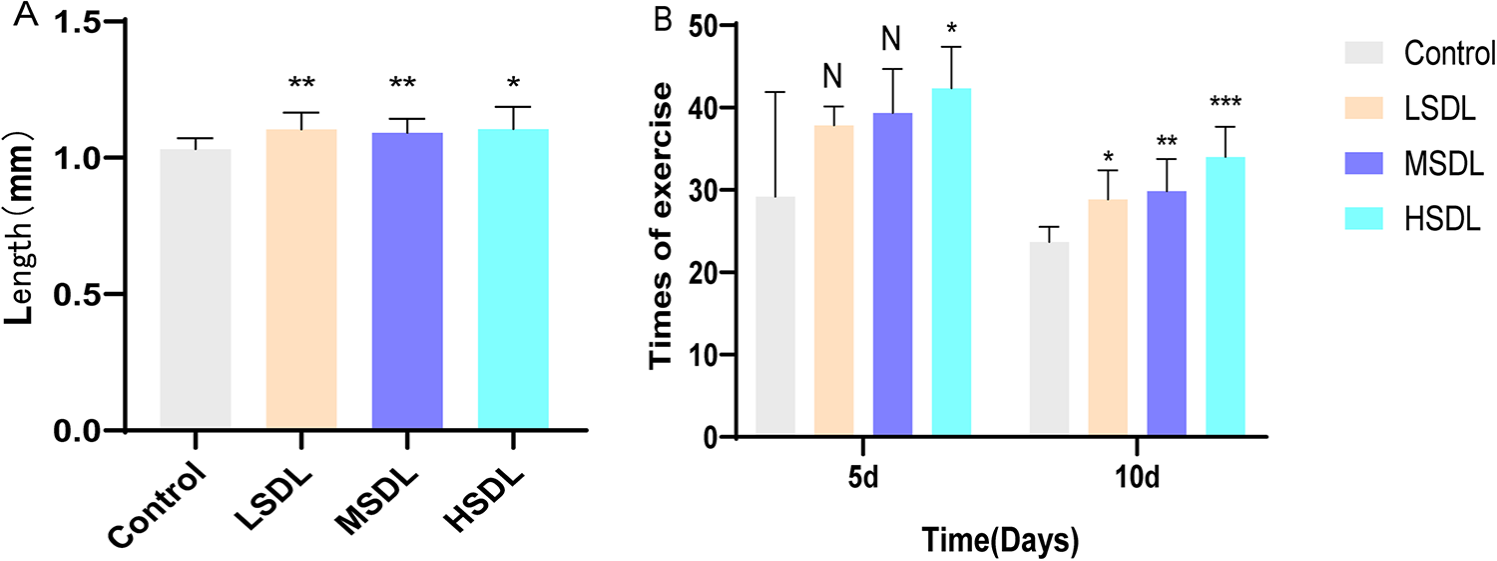
Effects of SDL on the body length and movement ability of *C. elegans* (**A**) different concentrations of SDL treatment on *C. elegans* have a certain increase in body length (**B**) the cultivation time is negatively correlated with the movement ability of *C. elegans*, and different concentrations of SDL can promote movement to a certain extent. ^N^*P*>0.05,*P < 0.05, ** *P* < 0.01 and *** *P* < 0.001 compared with Control group.

The growth of C. *elegans* can be visually reflected in body length. According to Fig. 9A, after 5 days of treatment, the body length of C. *elegans* in the blank control group was (1.03 ± 0.04) mm. Compared to the blank control group, the groups treated with SDL significantly promoted an increase in the body length of C. *elegans* (*P* < 0.05 or *P* < 0.01), with body lengths of (1.10 ± 0.06) mm, (1.09 ± 0.05) mm, and (1.11 ± 0.08) mm, respectively. The experimental results indicate that SDL have a certain promoting effect on the body length of C. *elegans*.

#### Determination of locomotor ability

The locomotor ability of nematodes reflects the function of their muscles, which is closely related to the aging process. With aging, the muscle contraction ability of nematodes gradually weakens, leading to phenomena such as uncoordinated movements, sluggishness, and even immobility. Therefore, the locomotor ability of nematodes reflects the activity level of their muscles and is an important indicator of whether nematodes are in a healthy state. As shown in Fig. 9B, with the extension of culture time, the number of body bends of nematodes significantly decreases. Compared to the blank control group, on the 5th day after administration, the nematodes in the HSDL group showed a significant increase in body bends (*P* < 0.05), with an improvement of 45.14%. On the 10th day after administration, both the SDL-treated groups could significantly increase the number of body bends of nematodes (*P* < 0.05), with the HSDL group showing the most significant improvement (*P* < 0.01), increasing the number of body bends by up to 43.66%.

#### Determination of lipofuscin content in nematodes

**Fig. 10:**
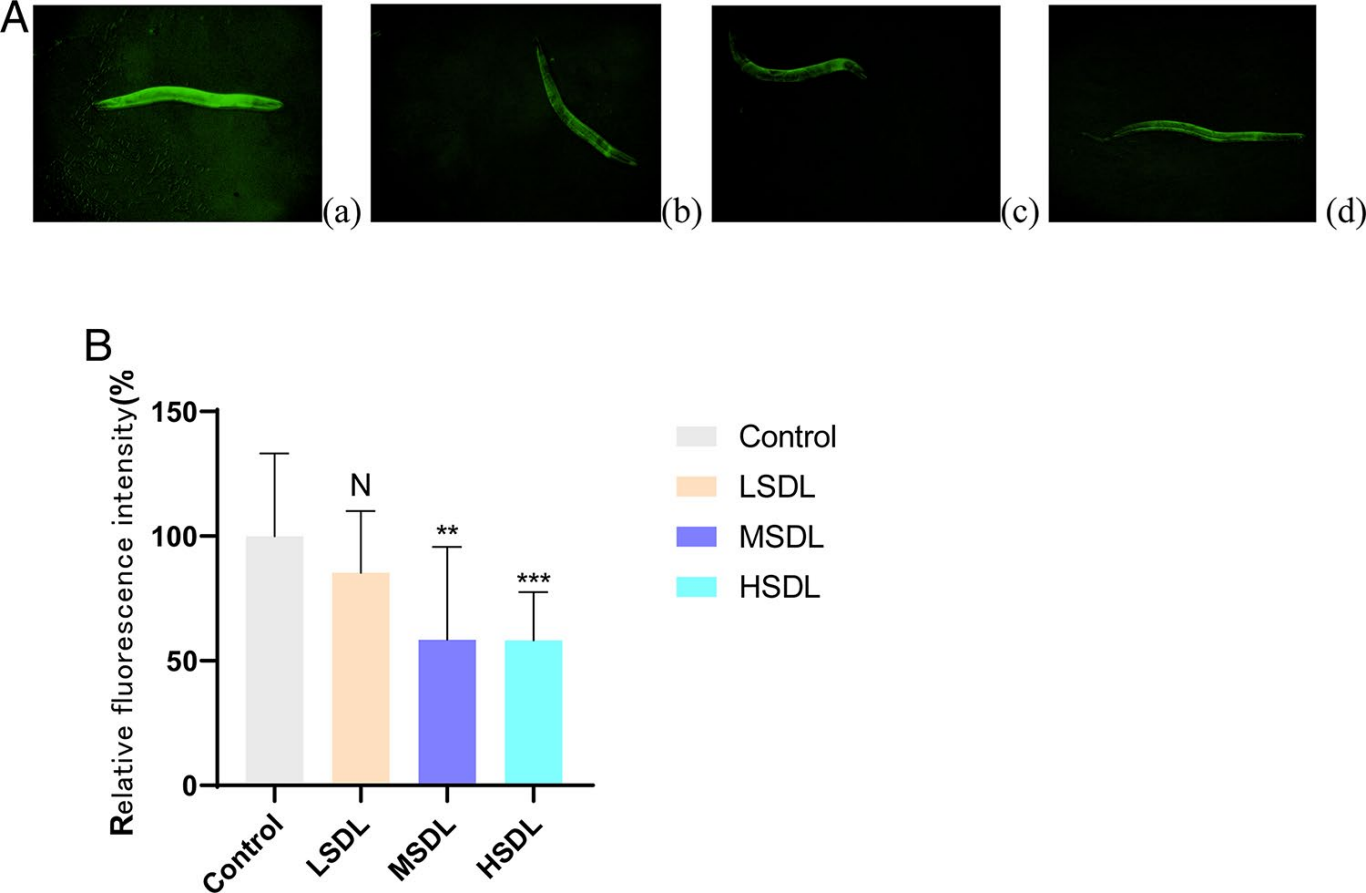
(**A**) Effects of SDL on the fluorescence intensity of lipofuscin in *C. elegans*(×10) (a. control group; b. LSDL group; c. MSDL group; d. HSDL group). (**B**) Effects of SDL on lipofuscin level of *C. elegans*. ^N^*P*>0.05, **P < 0.01 and ***P < 0.001 compared with Control group.

During the biological aging process, a large amount of highly oxidized and cross-linked proteins often appear, which are referred to as lipofuscin. It is considered a “hallmark dye” of aging and cannot be degraded by proteases and lysosomes. It has been found to exhibit autofluorescence in various aging animals and cells, including in nematodes^28^. As shown in Fig. 10A,B, compared to the blank control group, the nematodes treated with SDL showed a significant decrease in lipofuscin levels in their bodies (*P* < 0.05 or *P* < 0.01). Specifically, the MSDL and HSDL groups exhibited a reduction of 41.56% and 41.76%, respectively, compared to the blank control group. This indicates that SDL can to some extent reduce the level of lipofuscin in nematodes, thereby slowing down the aging process.

#### Heat stress experiment

**Fig. 11:**
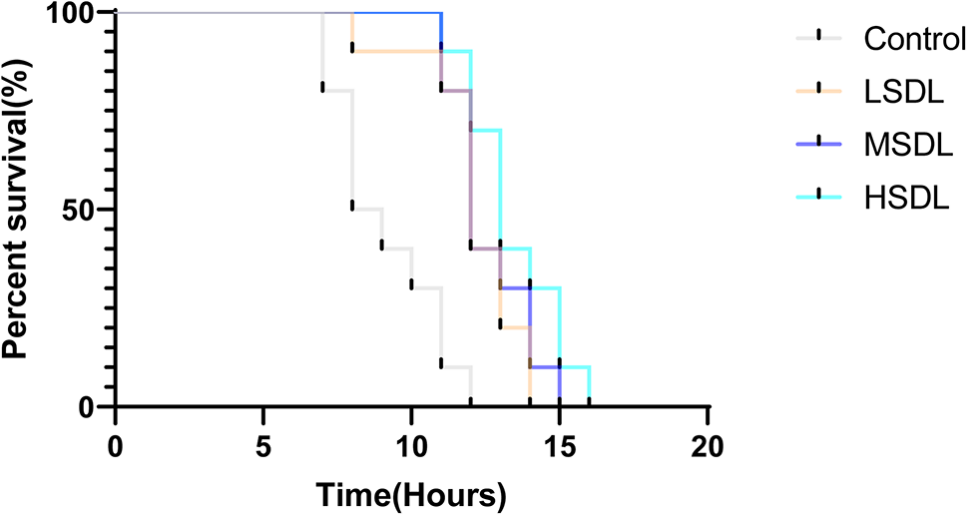
Effects of SDL on the lifespan of *C. elegans* under heat stress.

Heat stress can lead to heat shock and even death in nematodes, serving as an important trigger for oxidative damage in organisms. Research also suggests a positive correlation between the extension of nematode lifespan and resistance to heat stress^27^. As shown in Table 7; Fig. 11, compared to the blank control group, the nematodes treated with SDL at concentrations of 50, 100, and 200 µg/mL all exhibited a significant rightward shift in survival curves. The nematodes in the 200 µg/mL group showed an extension of survival time by up to 53.67%, with all differences being statistically significant (*P* < 0.001).

**Table 7:** Statistical analysis of *C. Elegans* survival time under heat stress.

Group	Dose(µg/mL)	The average lifespan /h	Increase percentage /%	The longest lifespan /h
Control	-	8.63 ± 0.40	-	11.67 ± 1.53
LSDL	50	11.83 ± 0.23***	37.07	14.00 ± 0.00
MSDL	100	12.13 ± 0.40***	40.54	15.00 ± 1.00
HSDL	200	13.27 ± 0.12***	53.67	15.33 ± 0.58

*P < 0.05, ** *P* < 0.01 and *** *P* < 0.001 compared with Control group.

## Conclusions

This study established a stable and feasible HPLC fingerprint of SDL samples. The overall pattern information of the HPLC fingerprint was statistically analyzed using multivariate methods such as cluster analysis, hierarchical cluster analysis (HCA), and principal component analysis (PCA), which comprehensively reflected the fingerprint and chemical characteristics. Therefore, the method developed in this study can distinguish differences in origin among SDL samples. The study also determined the content of four fatty acids (ALA, DHA, LA, OA) in SDL samples and compared the differences in component content among SDL samples from different origins. Thus, the combination of the HPLC fingerprint method with chemical pattern recognition and multi-component content determination can provide an initial assessment of the quality of SDL, offering a scientific basis for quality control of *Saposhnikovia divaricata*.

Furthermore, this study investigated the effect of SDL samples on delaying the aging of C. *elegans*. With increasing age and deepening aging of C. *elegans*, its lifespan shortens, growth ability weakens, muscle function deteriorates, and sensitivity to external stimuli decreases. Therefore, by measuring the lifespan, body length, head-tail swing frequency, and stress response of C. *elegans*, the aging process of C. *elegans* can be understood to some extent. Lipofuscin is often used as one of the indicators of aging. This study measured the above indicators, and the experimental results showed that SDL significantly extended the lifespan of C. *elegans*, promoted body growth, increased body swing frequency, enhanced heat stress response, and reduced the content of lipofuscin in C. *elegans*. These results demonstrate that SDL can improve the physical condition of C. *elegans*, delay the aging process, and provide theoretical references for research on the anti-aging effects of SDL.

## Data Availability

All data generated or analyzed during this study are included in this published article.

## Abbreviations

SDL: The lipophilic components of Saposhnikovia divaricata
ALA: Linolenic acid
DHA: Docosahexaenoic acid
LA: Linoleic acid
OA: Oleic acid
HPLC: High-performance liquid chromatography
PCA: Principal component analysis
HCA: Hierarchical cluster analysis
RSD: Relative standard deviation
